# Long-term boron-deficiency-responsive genes revealed by cDNA-AFLP differ between *Citrus sinensis* roots and leaves

**DOI:** 10.3389/fpls.2015.00585

**Published:** 2015-07-28

**Authors:** Yi-Bin Lu, Yi-Ping Qi, Lin-Tong Yang, Jinwook Lee, Peng Guo, Xin Ye, Meng-Yang Jia, Mei-Li Li, Li-Song Chen

**Affiliations:** ^1^Department of Resource and Environment, College of Resource and Environmental Science, Fujian Agriculture and Forestry UniversityFuzhou, China; ^2^Institute of Horticultural Plant Physiology, Biochemistry, and Molecular Biology, Fujian Agriculture and Forestry UniversityFuzhou, China; ^3^Institute of Materia Medica, Fujian Academy of Medical SciencesFuzhou, China; ^4^Department of Horticultural Science, Kyungpook National UniversityDaegu, South Korea; ^5^The Higher Educational Key Laboratory of Fujian Province for Soil Ecosystem Health and Regulation, Fujian Agriculture and Forestry UniversityFuzhou, China

**Keywords:** boron-deficiency, cDNA-AFLP, *Citrus sinensis*, leaves, roots, transcript-derived fragment

## Abstract

Seedlings of *Citrus sinensis* (L.) Osbeck were supplied with boron (B)-deficient (without H_3_BO_3_) or -sufficient (10 μM H_3_BO_3_) nutrient solution for 15 weeks. We identified 54 (38) and 38 (45) up (down)-regulated cDNA-AFLP bands (transcript-derived fragments, TDFs) from B-deficient leaves and roots, respectively. These TDFs were mainly involved in protein and amino acid metabolism, carbohydrate and energy metabolism, nucleic acid metabolism, cell transport, signal transduction, and stress response and defense. The majority of the differentially expressed TDFs were isolated only from B-deficient roots or leaves, only seven TDFs with the same GenBank ID were isolated from the both. In addition, ATP biosynthesis-related TDFs were induced in B-deficient roots, but unaffected in B-deficient leaves. Most of the differentially expressed TDFs associated with signal transduction and stress defense were down-regulated in roots, but up-regulated in leaves. TDFs related to protein ubiquitination and proteolysis were induced in B-deficient leaves except for one TDF, while only two down-regulated TDFs associated with ubiquitination were detected in B-deficient roots. Thus, many differences existed in long-term B-deficiency-responsive genes between roots and leaves. In conclusion, our findings provided a global picture of the differential responses occurring in B-deficient roots and leaves and revealed new insight into the different adaptive mechanisms of *C. sinensis* roots and leaves to B-deficiency at the transcriptional level.

## Introduction

Boron (B), as an essential micronutrient required for higher plants, is absorbed from soil solution by plant roots mainly in the form of boric acid. As boric acid in soils is easily leached under high rainfall conditions, B-deficient symptoms are often observed in many important agricultural crops, including citrus (Chen et al., 2012). According to investigation, a fair number of cultivated soils in southern and eastern China had very low concentration of hot water soluble B (less than 0.25 mg kg^−1^ DW) (Liu et al., 1989). Up to 45.5 and 9.0% of “Guanximiyou” pummelo (*Citrus grandis*) orchards in Pinghe, Zhangzhou, China were deficient in soil water-soluble B and leaf B, respectively (Huang et al., 2001).

In plants, B-deficiency affects many physiological and biochemical processes, such as gas exchange, nucleic acid metabolism, protein and amino acid (AA) biosynthesis, carbohydrate transport and accumulation, organic acid (OA) metabolism, cell division and elongation, cell wall structure, vascular development, phenolic metabolism, biosynthesis and transport of plant hormones, membrane integrity and oxidoreductase activity (Kouchi and Kumazawa, 1975; Tang and Dela Fuente, 1986; Han et al., 2008, 2009; Camacho-Cristóbal et al., 2008a; Chen et al., 2012; Hajiboland et al., 2012; Lu et al., 2014a).

B-deficiency causes remarkable alterations in the expression profiles of genes for various processes during plant growth and development, including cell wall modification (Camacho-Cristóbal et al., 2008b; Redondo-Nieto et al., 2012; Zhou et al., 2015), B uptake and translocation (Camacho-Cristóbal et al., 2008a), cell transport (Camacho-Cristóbal and González-Fontes, 2007; Redondo-Nieto et al., 2012; Zhou et al., 2015), vascular development (Yang et al., 2013a), stress response and defense (Redondo-Nieto et al., 2012; Zhou et al., 2015), protein and AA metabolism (Beato et al., 2010; Zhou et al., 2015), transcription, DNA metabolism, cell cycle, and signal transduction (Redondo-Nieto et al., 2012) in tobacco roots, leaves, and cells, citrus roots, and leaf veins, *Medicago truncatula* root nodules, *Arabidopsis* roots, shoots, and seedlings. Most research, however, has focused on roots (Camacho-Cristóbal et al., 2011), because the inhibition of root growth is one of the most rapid responses of plants to B-deficiency (Bohnsack and Albert, 1977). Less is known about the effects of B-deficiency on leaf transcriptomics. Also, limited data are available on B-deficiency-responsive genes in woody plants.

There are several comparative studies showing that the effects of B-deficiency on gas exchange, carbohydrates and OAs and related metabolic enzymes, nitrogen and phenolic metabolisms differ between roots and leaves (Stavrianakou et al., 2006; Camacho-Cristóbal et al., 2008a; Hajiboland et al., 2013; Lu et al., 2014a). Thus, B-deficiency-induced changes in transcriptomics should be different between roots and leaves. To our knowledge, such data are not yet available in woody plants.

In this study, we first compared B-deficiency-responsive genes in *Citrus sinensis* roots and leaves using cDNA-amplified fragment length polymorphism (cDNA-AFLP) in order to (*a*) determine the mechanisms of plants to deal with B-deficiency at the transcriptional level and (*b*) understand the differences in B-deficiency-induced alterations in gene expression between roots and leaves.

## Materials and methods

### Plant materials

This study was conducted at Fujian Agriculture and Forestry University, Fuzhou, China (26°5′ N, 119°14′ E). “Xuegan” (*Citrus sinensis*) seedlings were used in this study, because *C. sinensis* is polyembryonic seed development, an apomictic process in which many embryos are initiated directly from the maternal nucellar cells surrounding the embryo sac containing a developing zygotic embryo (Aleza et al., 2010).

### Experimental design

Plant culture and B-treatments were performed according to Yang et al. (2013b). In late-May (5 weeks after germination), uniform seedlings were transplanted into 6 L pots (two per pot) containing river sand and grown in a greenhouse under natural photoperiod. Ten weeks after transplanting, each pot were irrigated every other day until dripping with B-deficient (without H_3_BO_3_) or -sufficient (10 μM H_3_BO_3_) nutrient solution for 15 weeks. There were 20 pots per treatment in a completely randomized design. At the end of the experiment, fully expanded (about 7-week-old) leaves (midribs and petioles removed) and ca. 5-mm-long root apices were collected at noon under full sun from different replicates and treatments and immediately frozen in liquid N_2_. Both leaf and root samples were stored at −80°C until they were used for cDNA-AFLP and qRT-PCR analysis. The remaining seedlings that were not sampled were used to measure root, stem, and leaf dry weights (DWs) and root and leaf concentration of B.

### Measurements of root, stem, and leaf DWs, and root and leaf B concentration

Ten plants per treatment from different replicates were harvested and divided into roots, stems and leaves. After being dried 70°C for 48 h, their DWs were weighted.

For the determination of B concentration, about 7-week-old leaves (midribs and petioles removed) and fibrous roots were collected and dried at 70°C for 48 h. Dried samples were ground in a mortar to pass a 40-mesh sieve, then ashed at 500°C for 5 h, finally dissolved in 0.1 M HCl. B concentration in the solution was assayed by the modified curcumin method (Kowalenko and Lavkulich, 1976). There were six replicates per treatment.

### RNA preparation, cDNA synthesis and CDNA-AFLP analysis

Equal amounts of frozen leaf (root) samples collected from five plants (one per pot) were mixed as a biological replicate. There were three biological replicates for each treatment. Total RNA was independently extracted three times from the frozen samples using Recalcitrant Plant Total RNA Extraction Kit (Centrifugal column type, Bioteke Corporation, China) according to manufacturer's instructions. cDNA synthesis and cDNA-AFLP analysis were performed according to Zhou et al. (2013). After the integrity and quantity of total RNA being checked, first-strand cDNA was synthesized. The resulting double-stranded cDNA was purified using equal volume of phenol: chloroform: isoamyl alcohol (25: 24: 1). Double-stranded cDNA (600 ng) was digested with restriction enzymes: 5 U each of *Eco*R I (Thermo Scientific, Massachusetts, USA; 3 h at 37°C) and *Mse* I (*Tru*1I, Thermo Scientific, Massachusetts, USA; 3 h at 65°C). The resulting restricted fragments were ligated to adaptors (*EcoR* I, 0.2 μM forward primer: 5′- CTCGTAGACTGC GTACC-3′ and reverse primer: 3′- CATCTG ACGCATGGTTAAP -5′; *Mse* I, 2 μM forward primer: 5′-GACGAT GAGTCCTGAG-3′ and reverse primer: 3′-TA CTCAGGACTCATP-5′) with T4-DNA ligase (Thermo Scientific, Massachusetts, USA) for 10–16 h at 16°C. The resulting ligated products were pre-amplified with the corresponding pre-amplification primers: *Eco*R I, 5′-GACTGCGATCCAATTC-3′ and *Mse* I, 5′-GATGAG TCCTGAGTAA-3′. From a 100-fold dilution of the pre-amplified samples, a 5 μL diluted sample was used for the selective amplification using 256 combinations of the following primers: 16 derivatives of *Eco*R I primers 5′-GACTGCGATCCAATT CEE-3′ and 16 derivatives of *Mse* I primers 5′-GATGAG TCCTGAGTAAMM-3′; where EE and MM represented AA, AT, AC, AG, TA, TC, TT, TG, CA, CT, CG, CC, GA, GC, GT, and GG. The selective amplification products were separated on a 6% (w/v) polyacrylamide gel run at 50 W for 2.5 h. The gels were silver stained to visualize the cDNA bands. Samples for cDNA-AFLP analysis were run in three replicates at least.

The up- or down-regulated cDNA-AFLP bands (transcript-derived fragments, TDFs) were selected based on their presence, absence, or differential intensity and cut out with a scalpel, and incubated in 50 μL of dd H_2_O for 30 min in a boiling water bath, then centrifuged at 10000 rpm (Eppendorf 5418R, Hamburg, Germany). The supernatant (eluted DNA) was re-amplified by PCR using the same primer combinations. The re-amplified products were checked on 1% (w/v) agarose gels, each band was isolated and eluted using DNA Agarose Gel Recovery Kit (Solarbio, China). Before being sequenced by BGI Technology Corporation (Shenzhen, China), these TDFs fragments were ligated to pGEM-T EASY vector according to usage information of pGEM®-T Easy Vector System I (Promega, USA), then transduced into *Escherichia coli* (DH5α) competent cells using ampicillin as the selecting agent. All sequences were input into the VecScreen (http://www.ncbi.nlm.nih.gov/VecScreen/VecScreen) to identify and remove all of the vector sequence. Homology of TDFs' sequences was analyzed using the BLASTX and BLASTN searching engines (http://www.blast.ncbi.nlm.nih.gov/Blast). Their functional categories were assigned based on the analysis of information reported for each sequence by The Gene Ontology (http://amigo.geneontology.org/cgi-bin/amigo/blast) and Uniprot (http://www.uniprot.org/).

### qRT-PCR analysis

Both sample collecting and total RNA extraction were performed as described above. qRT-PCR analysis was performed according to Zhou et al. (2013). Specific primers were designed from the sequences of 51 differentially expressed TDFs using Primer Premier Version 5.0 (PREMIER Biosoft International, CA, USA). The sequences of the F and R primers used were listed in Table S1. Samples for qRT-PCR were run in three biological replicates with two technical replicates. Relative gene expression was calculated using ddCt algorithm. For the normalization of gene, citrus *actin* (GU911361.1) was used as an internal standard and the sample from B-sufficient treated plants was used as reference sample, which was set to 1.

### Statistical analysis

Results represented the mean ± SD. Statistical analyses of data were carried out by unpaired *t*-test at *P* < 0.05 level.

## Results

### Plant growth and B concentration in roots and leaves

Seedlings treated without H_3_BO_3_ had slower growth and less leaf and root level of B than those treated with 10 μM H_3_BO_3_ (Figure S1). B concentration in leaves from seedlings treated without H_3_BO_3_ was lower than the sufficient range of 30–100 μg g^−1^ DW (Chapman, 1968). Also, a typical B-deficient symptom (i.e., corky split veins) was observed in leaves from seedlings treated without H_3_BO_3_ (Han et al., 2008). Thus, seedlings treated without H_3_BO_3_ are considered B-deficient, and those treated with 10 μM H_3_BO_3_ are considered B-sufficient.

### Differentially expressed genes in B-deficient roots and leaves

A total of 256 selective primer combinations were used to isolate the differentially expressed TDFs from B-deficient roots and leaves. Figure S2 displayed the typical picture of a silver-stained cDNA-AFLP gel. We amplified a total of 5247 (5579) reproducible, clear, and unambiguous cDNA-AFLP bands (TDFs) from B-deficient roots (leaves), with an average of 20.5 (21.8) TDFs in roots (leaves) for each primer combination. A TDF with both a *P*-value of less than 0.05 and an average fold change of more than 1.5 was considered differentially expressed. Here, 131 and 165 differentially expressed and reproducible TDFs were obtained from B-deficient roots and leaves, respectively. After all these TDFs were reamplified, cloned, and sequenced, 114 and 129 TDFs from roots and leaves produced useable sequence data. All these data were blasted against the sequence data available in GenBank. Eighty-three root TDFs and 92 leaf TDFs showed significant homology to genes encoding known, putative uncharacterized, hypothetical and unknown proteins, and the remaining 31 root TDFs and 37 leaf TDFs did not share homologous with any nucleotide or AA sequence in the public databases.

### Functions of the differentially expressed genes in roots and leaves

Among the 83 matched root TDFs, 38 TDFs were up-regulated and 45 TDFs were down-regulated by B-deficiency. According to the biological properties, these TDFs were associated with carbohydrate and energy metabolism (11), nucleic acid metabolism (13), protein and AA metabolism (10), cell transport (9), signal transduction (7), stress response and defense (8), lipid metabolism (4), cell wall modification (2), and others (19) (Table 1; Figure S3). For the 92 matched leaf TDFs, 54 TDFs were increased and 38 TDFs were decreased by B-deficiency. These TDFs were classified into the following categories: carbohydrate and energy metabolism (12), nucleic acid metabolism (11), protein and AA metabolism (19), cell transport (10), signal transduction (5), stress response and defense (8), lipid metabolism (6), cell wall modification (1), and others (20) (Table 2; Figure S3). As shown in Figure S3, the majority of the differentially expressed TDFs were isolated only from B-deficient roots or leaves, only seven TDFs with the same GenBank ID were isolated from the both.

**Table 1:** **Homologies of differentially expressed cDNA-AFLP fragments with known gene sequences in database using BLASTN algorithm along with their expression patterns in B-deficient ***Citrus sinensis*** roots**.

**TDF#**	**Size (bp)**	**Homologous protein**	**Organism origin**	**GenBank ID**	***E*-value**	**Identity**	**Fold change**
**GENES INVOLVED IN CARBOHYDRATE AND ENERGY METABOLISM**							
R63-3b	220	UDP-glycosyltransferase 83A1-like	*Citrus sinensis*	XP_006477640.1	3.00E-41	94%	0
R242-1b	264	UDP-glucosyl transferase 73C1	*Arabidopsis thaliana*	NP_181213.1	0.66	45%	0
R244-2b	264	UDP-glucosyl transferase 73C1	*Arabidopsis thaliana*	NP_181213.1	0.66	45%	0.61 ± 0.04
R255-1b	264	UDP-glycosyltransferase 73C4-like	*Cucumis sativus*	XP_004151569.1	1.00E-07	89%	0
R256-2b	264	UDP-glycosyltransferase 73C4-like	*Cucumis sativus*	XP_004151569.1	1.00E-07	89%	0
**R132-6a**	**185**	**Cytochrome P450**	***Medicago truncatula***	**XP_003636948.1**	**2.00E-17**	**60%**	+
R167-3b	184	Pyruvate kinase	*Medicago truncatula*	XP_003611452.1	1.00E-06	83%	0
R79-1b	257	NAD-dependent malic enzyme 59 kDa isoform, mitochondrial	*Citrus sinensis*	XP_006491046.1	8.00E-41	85%	0
**R59-3a**	**161**	**Fumarate hydratase 2**	***Arabidopsis thaliana***	**NP_001119412.1**	**5.00E-19**	**93%**	+
**R3-1a**	**244**	**ATP synthase subunit beta**	***Medicago truncatula***	**XP_003627732.1**	**1.00E-22**	**66%**	**2.90** ± **0.20**
**R16-2a**	**271**	**ATP synthase subunit beta**	***Medicago truncatula***	**XP_003627732.1**	**7.00E-09**	**86%**	+
**GENES INVOLVED IN NUCLEIC ACID METABOLISM**							
**R23-2a**	**198**	**Heat shock factor protein HSF24-like**	***Citrus sinensis***	**XP_006466606.1**	**4.00E-30**	**97%**	**5.68** ± **0.50**
**R15_1a**	**214**	**Zinc finger protein 4**	***Theobroma cacao***	**XP_007045479.1**	**4.00E-13**	**64%**	+
**R198-1a**	**262**	**Protein vip1-like isoform X1**	***Citrus sinensis***	**XP_006482580.1**	**1.00E-12**	**97%**	+
**R13-2a**	**434**	**U2 small nuclear ribonucleoprotein A'-like**	***Citrus sinensis***	**XP_006487666.1**	**5.00E-43**	**100%**	+
**R157-1a**	**256**	**Heterogeneous nuclear ribonucleoproteins A1 homolog isoform X1**	***Citrus sinensis***	**XP_006485663.1**	**9.00E-45**	**99%**	+
**R105-2a**	**300**	**RRNA intron-encoded homing endonuclease**	***Medicago truncatula***	**XP_003614387.1**	**1.00E-12**	**92%**	**5.98** ± **0.10**
**R195-1a**	**296**	**Cyclin-like family protein isoform 2**	***Theobroma cacao***	**XP_007028689.1**	**1.00E-53**	**97%**	**9.77** ± **0.98**
**R147-4a**	**414**	**Cyclic dof factor 2-like**	***Citrus sinensis***	**XP_006482609.1**	**1.00E-61**	**90%**	+
R81-3b	149	Pre-mRNA-splicing factor SYF1-like	*Citrus sinensis*	XP_006467884.1	6.00E-19	98%	0.52 ± 0.04
R157-4b	218	RNA polymerase II, Rpb4, core protein	*Arabidopsis thaliana*	NP_196554.1	2.00E-24	86%	0
R247-1b	248	MuDR family transposase isoform 2	*Theobroma cacao*	XP_007031260.1	4.00E-38	87%	0
R164-1b	244	Transcription factor TCP9-like	*Citrus sinensis*	XP_006477362.1	6.00E-10	97%	0
R100-3b	278	Agamous-like MADS-box protein AGL92	*Arabidopsis thaliana*	NP_174445.1	4.40	40%	0
**GENES INVOLVED IN PROTEIN AND AMINO ACID METABOLISM**							
R100-2b	301	60S ribosomal protein L29-1-like	*Citrus sinensis*	XP_006488862.1	1.00E-32	98%	0
R174-1b	229	60S ribosomal protein L31-like	*Citrus sinensis*	XP_006482903.1	4.00E-20	98%	0
R253-3b	164	Elongation factor G, chloroplastic-like	*Citrus sinensis*	XP_006477256.1	8.00E-25	94%	0
R256-1b	302	Elongation factor Tu,chloroplastic-like	*Citrus sinensis*	XP_006476894.1	4.00E-04	88%	0.24 ± 0.05
**R63-1a**	**240**	**Ribosomal protein S3**	***Citrus sinensis***	**YP_740514.1**	**3.00E-33**	**95%**	+
**R103-3a**	**265**	**Asx tRNA synthetase (AspRS/AsnRS) class II core domain-contating protein**	***Arabidopsis thaliana***	**NP_849558.1**	**2.00E-32**	**86%**	+
R35-2b	363	F-box protein At4g18380-like	*Citrus sinensis*	XP_006470730.1	8.00E-22	100%	0
R112-5b	161	BTB/POZ domain-containing protein At5g41330-like	*Citrus sinensis*	XP_006476542.1	5.00E-19	98%	0.40 ± 0.01
**R132-3a**	**276**	***Chain A, crystal structure off ll-diaminopimelate aminotransferase from Arabidopsis thaliana complexed with l-malate ion***	***Arabidopsis thaliana***	**2Z1Z_A**	**6.00E-46**	**85%**	+
**R104-1a**	**262**	**Bifunctional aspartokinase/homoserine dehydrogenase 1, chloroplastic-like**	***Citrus sinensis***	**XP_006478426.1**	**1.00E-46**	**100%**	+
**GENES INVOLVED IN CELL TRANSPORT**							
**R23-1a**	**282**	**Putative vacuolar protein sorting-associated protein 13B-like isoform X1**	***Citrus sinensis***	**XP_006492899.1**	**2.00E-38**	**97%**	+
**R88-6a**	**138**	**Peroxisomal membrane ABC transporter family, PMP family isoform 2**	***Theobroma cacao***	**XP_007047973.1**	**0.001**	**72%**	**9.76** ± **1.05**
**R132-4a**	**239**	**Calcium-binding mitochondrial carrier protein SCaMC-1-like**	***Citrus sinensis***	**XP_006485062.1**	**2.00E-37**	**98%**	+
**R210-1a**	**282**	**Early-responsive to dehydration stress protein (ERD4)**	***Arabidopsis thaliana***	**NP_564354.1**	**7.00E-18**	**59%**	**16.62** ± **1.04**
R243-1b	341	Bet1-like SNARE protein 1-1	*Medicago truncatula*	KEH42573.1	3.00E-42	83%	0
R253-2b	327	Major facilitator superfamily protein	*Theobroma cacao*	XP_007027453.1	3.00E-20	56%	0.05 ± 0.01
R114-1b	243	Brefeldin A-inhibited guanine nucleotide-exchange protein 5-like isoform X1	*Citrus sinensis*	XP_006474544.1	5.00E-39	96%	0.52 ± 0.04
R209-2b	232	Brefeldin A-inhibited guanine nucleotide-exchange protein 5-like isoform X1	*Citrus sinensis*	XP_006474544.1	6.00E-38	97%	0.38 ± 0.04
R117-1b	297	Transmembrane emp24 domain-containing protein	*Arabidopsis thaliana*	NP_564256.1	9.00E-37	62%	0.21 ± 0.03
**GENES INVOLVED IN SIGNAL TRANSDUCTION**							
R170-1b	296	Probable receptor-like protein kinase At1g11050-like isoform X1	*Citrus sinensis*	XP_006471114.1	1.00E-53	97%	0
R219-2b	238	Serine/threonine-protein kinase cx32 isoform 2	*Theobroma cacao*	XP_007043058.1	8.00E-37	90%	0
R251-3b	245	Calcium and calcium/calmodulin-dependent serine/threonine-protein kinase isoform 1	*Theobroma cacao*	XP_007028376.1	2.00E-39	90%	0
R119-1b	257	Auxin-responsive protein IAA13, putative isoform 1	*Theobroma cacao*	XP_007035277.1	3.00E-31	71%	0.30 ± 0.02
R243-2b	312	Phosphatidylinositol 4-kinase gamma 7-like	*Citrus sinensis*	XP_006484264.1	2.00E-30	97%	0.45 ± 0.03
R182-1b	336	Fasciclin-like arabinogalactan protein 4-like	*Citrus sinensis*	XP_006495462.1	2.00E-47	99%	0.29 ± 0.05
R67-2b	259	Rae1-like protein At1g80670-like isoform X1	*Citrus sinensis*	XP_006465252.1	2.00E-49	99%	0.28 ± 0.01
**GENES INVOLVED IN STRESS RESPONSE AND DEFENSE**							
**R100-1a**	**332**	**Plant senescence-associated protein**	***Theobroma cacao***	**XP_007099561.1**	**1.00E-40**	**93%**	+
**R148-4a**	**248**	**Putative senescence-associated protein**	***Pisum sativum***	**BAB33421.1**	**1.00E-18**	**66%**	+
**R57-1a**	**205**	**Peroxidase 30-like isoform X2**	***Citrus sinensis***	**XP_006469710.1**	**5.00E-33**	**98%**	+
R186-6b	207	Thioredoxin M4,chloroplastic-like isoform X1	*Citrus sinensis*	XP_006464740.1	5.00E-18	98%	0
R178-4b	225	Thiosulfate sulfurtransferase 18-like isoform X1	*Citrus sinensis*	XP_006466965.1	4.00E-41	92%	0
R169-1b	258	Serine hydroxymethyltransferase 7-like	*Citrus sinensis*	XP_006472687.1	1.00E-38	99%	0
R68-1b	258	Putative disease resistance protein RGA3-like isoform X2	*Citrus sinensis*	XP_006471965.1	1.00E-20	98%	0.55 ± 0.07
R112-3b	213	Putative disease resistance protein RGA4-like	*Citrus sinensis*	XP_006472380.1	4.00E-28	92%	0.18 ± 0.01
**GENES INVOLVED IN LIPID METABOLISM**							
R118-2b	260	Cytochrome P450 86B1-like	*Citrus sinensis*	XP_006484536.1	1.00E-29	96%	0
R60-1b	239	3-ketoacyl-CoA synthase 10-like	*Citrus sinensis*	XP_006474655.1	1.00E-41	97%	0.22 ± 0.04
**R190-1a**	**177**	**Beta-hydroxyacyl-ACP dehydratase family protein**	***Populus trichocarpa***	**XP_002304073.2**	**0.005**	**54%**	+
**R132-5a**	**201**	**Non-lysosomal glucosylceramidase-like isoform X2**	***Citrus sinensis***	**XP_006493710.1**	**9.00E-32**	**98%**	+
**CELL WALL MODIFICATION**							
**R63-2a**	**231**	**14 kDa proline-rich protein DC2.15-like**	***Citrus sinensis***	**XP_006477234.1**	**2.00E-20**	**88%**	+
R55-1b	206	Expansin-like B1-like	*Citrus sinensis*	XP_006470179.1	1.00E-18	97%	0.43 ± 0.07
**OTHERS**							
R103-1b	302	3-oxo-Delta(4,5)-steroid 5-beta-reductase-like isoform X2	*Citrus sinensis*	XP_006471285.1	1.00E-54	99%	0
**R64-4a**	**253**	**Gibberellin 2-beta-dioxygenase 2-like**	***Citrus sinensis***	**XP_006492455.1**	**2.00E-39**	**100%**	+
R211-1b	242	Cox19-like CHCH family protein	*Theobroma cacao*	XP_007042812.1	5.00E-18	73%	0.23 ± 0.06
R240-2b	203	LOW QUALITY PROTEIN: reticuline oxidase-like protein-like	*Cucumis sativus*	XP_004162849.1	3.00E-26	75%	0
**R205-5a**	**223**	**Mitochondrial protein, putative**	***Medicago truncatula***	**XP_003588355.1**	**3.00E-04**	**88%**	+
**R188-2a**	**251**	**Plasma membrane isoform 4**	***Theobroma cacao***	**XP_007029644.1**	**8.00E-22**	**73%**	+
**R105-1a**	**307**	**Probable methyltransferase PMT9-like**	***Citrus sinensis***	**XP_006470210.1**	**2.00E-48**	**95%**	**6.70** ± **0.98**
**R129-2a**	**213**	**Citrus dioxygenase**	***Citrus limetta***	**AER36089.1**	**6.00E-35**	**92%**	**2.50** ± **0.10**
**R67-1a**	**322**	**Uncharacterized protein LOC102615853**	***Citrus sinensis***	**XP_006488831.1**	**2.00E-49**	**99%**	**6.70** ± **1.01**
**R129-3a**	**209**	**Uncharacterized protein LOC102615510**	***Citrus sinensis***	**XP_006480156.1**	**2.00E-28**	**96%**	**9.89**
**R143-2a**	**227**	**Hypothetical protein CICLE_v10022514mg**	***Citrus clementina***	**XP_006444049.1**	**5.00E-23**	**100%**	**3.78** ± **0.25**
**R215-1a**	**246**	**Hypothetical protein CICLE_v10018848mg**	***Citrus clementina***	**XP_006439810.1**	**2.00E-14**	**97%**	+
**R209-6a**	**296**	**Unknown**	***Zea mays***	**ACR36970.1**	**2.00E-53**	**97%**	+
**R20-1a**	**296**	**Hypothetical protein**	***Arabidopsis thaliana***	**BAF01964.1**	**3.00E-48**	**100%**	**3.20** ± **0.30**
**R82-7a**	**296**	**Hypothetical protein**	***Arabidopsis thaliana***	**BAF01964.1**	**1.00E-54**	**100%**	**9.83** ± **1.22**
R26-1b	110	Uncharacterized serine-rich protein C215.13-like	*Citrus sinensis*	XP_006478568.1	2.00E-08	89%	0
R57-4b	378	Uncharacterized protein	*Arabidopsis thaliana*	NP_001118287.1	0.16	64%	0.50 ± 0.04
R251-1b	281	Uncharacterized membrane protein YMR155W-like	*Citrus sinensis*	XP_006491715.1	4.00E-20	62%	0
R51-1b	212	Hypothetical protein CISIN 1g029245	*Citrus sinensis*	KDO43856.1	3.00E-35	90%	0.199 ± 0.05

*Data are means ± SD (n = 3); Expression ratio, 0 means TDFs were only detected in control roots; + means TDF were only detected in the B-deficient roots. #, Number; Functional classification was performed based on the information reported for each sequence by The Gene Ontology (http://amigo1.geneontology.org/cgi-bin/amigo/blast.cgi) and Uniprot (http://www.uniprot.org/). Relative expression ratio was obtained by gel image analysis, which was performed with PDQuest version 8.0.1 (Bio-Rad, Hercules, CA, USA). Up-regulated TDFs were highlighted in bold*.

**Table 2:** **Homologies of differentially expressed cDNA-AFLP fragments with known gene sequences in database using BLASTN algorithm along with their expression patterns in B-deficient ***Citrus sinensis*** leaves**.

**TDF#**	**Size (bp)**	**Homologous protein**	**Organism origin**	**GenBank ID**	***E*-value**	**Identity**	**Fold change**
**GENES INVOLVED IN CARBOHYDRATE AND ENERGY METABOLISM**							
**L3-2a**	**225**	**UDP-glycosyltransferase 74E2-like**	***Citrus sinensis***	**XP_006469356.1**	**2.00E-37**	**96%**	**3.98** ± **0.02**
**L201-1a**	**245**	**UDP-glycosyltransferase 84B2-like**	***Citrus sinensis***	**XP_006486998.1**	**3.00E-32**	**95%**	+
**L29-2a**	**174**	**UDP-arabinose 4-epimerase 1-like isoform X1**	***Citrus sinensis***	**XP_010478486.1**	**2.00E-26**	**100%**	**5.59** ± **0.10**
L255-2b	367	Enolase-like	*Citrus sinensis*	XP_006481907.1	3.00E-61	93%	0
L256-2b	367	Enolase-like	*Citrus sinensis*	XP_006481907.1	3.00E-62	93%	0
L63-1b	536	Chlorophyll a-b binding protein 6A, chloroplastic-like	*Fragaria vesca subsp. vesca*	XP_004307395.1	9.00E-89	89%	0
L191-2b	280	Ribulose bisphosphate carboxylase small chain, chloroplastic-like isoform X1	*Citrus sinensis*	XP_006482254.1	1.00E-54	98%	0
**L27-2a**	**608**	**Protochlorophyllide reductase, chloroplastic-like**	***Citrus sinensis***	**XP_006464717.1**	**9.00E-126**	**98%**	+
**L55-3a**	**608**	**Protochlorophyllide reductase, chloroplastic-like**	***Citrus sinensis***	**XP_006464717.1**	**2.00E-122**	**96%**	+
**L199-1a**	**254**	**Magnesium-chelatase subunit ChlH, chloroplastic-like**	***Citrus sinensis***	**XP_006489988.1**	**2.00E-42**	**97%**	+
L241-5b	201	Cytosolic endo-beta-N-acetylglucosaminidase-like	*Citrus sinensis*	XP_006486257.1	4.00E-21	98%	0.09 ± 0.02
**L64-1a**	**309**	**Obg-like ATPase 1-like**	***Citrus sinensis***	**XP_006468322.1**	**9.00E-58**	**99%**	+
**GENES INVOLVED IN NUCLEIC ACID METABOLISM**							
**L19-1a**	**235**	**Putative DEAD-box ATP-dependent RNA helicase 33-like**	***Citrus sinensis***	**XP_006477444.1**	**8.00E-25**	**97%**	**9.54** ± **1.19**
**L67-1a**	**235**	**Putative DEAD-box ATP-dependent RNA helicase 33-like**	***Citrus sinensis***	**XP_006477444.1**	**8.00E-25**	**97%**	+
**L194-3a**	**201**	**DEAD-box ATP-dependent RNA helicase 39-like**	***Citrus sinensis***	**XP_006473146.1**	**4.00E-09**	**98%**	+
**L253-7a**	**200**	**DEAD-box ATP-dependent RNA helicase 27-like**	***Citrus sinensis***	**XP_006471112.1**	**3.00E-31**	**97%**	**5.04** ± **0.99**
**L235-1a**	**293**	**WD40 repeat-containing protein SMU1-like**	***Citrus sinensis***	**XP_006474451.1**	**5.00E-53**	**99%**	**3.45** ± **0.01**
**L116-1a**	**236**	**Zinc finger protein, putative isoform 2**	***Arabidopsis thaliana***	**XP_007013705.1**	**3.00E-18**	**89%**	+
**L184-1a**	**150**	**Putative replication factor A**	***Arabidopsis thaliana***	**AAF26967.1**	**0.014**	**68%**	**10.01** ± **2.18**
L253-3b	277	RNA binding protein HCF152	*Arabidopsis thaliana*	NP_187576.1	2.00E-30	79%	0.32 ± 0.03
L175-1b	296	Methyl-CpG-binding domain-containing protein 9-like isoform X2	*Theobroma cacao*	XP_006483833.1	4.00E-53	96%	0
L239-3b	260	Pentatricopeptide repeat-containing protein At4g20770-like	*Citrus sinensis*	XP_006468369.1	2.00E-46	99%	0
L227-1b	251	Homeobox protein 40	*Arabidopsis thaliana*	NP_195392.2	6.00E-23	71%	0
**GENES INVOLVED IN PROTEIN AND AMINO ACID METABOLISM**							
**L132-2a**	**218**	**40S ribosomal protein S8 isoform 1**	***Theobroma cacao***	**XP_007049644.1**	**3.00E-36**	**97%**	+
**L100-3a**	**243**	**60S ribosomal protein L29-1-like**	***Citrus sinensis***	**XP_006488862.1**	**2.00E-32**	**97%**	+
**L209-1a**	**333**	**Translation initiation factor IF-3**	***Arabidopsis thaliana***	**NP_174696.2**	**1.00E-15**	**47%**	**7.77** ± **0.13**
**L221-1a**	**278**	**Elongation factor 2-like**	***Cucumis sativus***	**XP_004173402.1**	**9.00E-53**	**91%**	**6.60** ± **0.09**
L253-4b	264	Protein disulfide isomerase-like 1-4-like isoform X1	*Citrus sinensis*	XP_006488102.1	2.00E-41	95%	0
L209-3b	253	Ribosome biogenesis protein BMS1 homolog isoform X1	*Citrus sinensis*	XP_006487977.1	1.00E-42	100%	0
L242-2b	302	Elongation factor Tu, chloroplastic-like	*Citrus sinensis*	XP_006476894.1	2.00E-04	91%	0
L256-1b	302	Elongation factor Tu, chloroplastic-like	*Citrus sinensis*	XP_006476894.1	4.00E-04	88%	0
**L113-1a**	**336**	**Proteasome subunit beta type-4-like**	***Citrus sinensis***	**XP_006488144.1**	**2.00E-37**	**98%**	**7.98** ± **0.21**
**L215-1a**	**270**	**U-box domain-containing protein 33**	***Arabidopsis thaliana***	**NP_182115.2**	**1.00E-23**	**57%**	**6.63** ± **0.34**
**L52-3a**	**556**	**Tubby-like F-box protein 5-like isoform X2**	***Citrus sinensis***	**XP_006473523.1**	**2.00E-111**	**99%**	+
**L61-1a**	**556**	**Tubby-like F-box protein 5-like isoform X2**	***Citrus sinensis***	**XP_006473523.1**	**2.00E-111**	**99%**	+
**L253-2a**	**303**	**Aminoacylase-1-like isoform X2**	***Citrus sinensis***	**XP_006486586.1**	**4.00E-38**	**92%**	+
**L82-2a**	**125**	**ATP-dependent zinc metalloprotease FTSH 2, chloroplastic-like**	***Citrus sinensis***	**XP_006468976.1**	**0.26**	**88%**	+
**L211-1a**	**391**	**COP9 signalosome complex subunit 1**	***Theobroma cacao***	**XP_007051916.1**	**5.00E-23**	**59%**	+
**L249-3a**	**167**	**Auxin-resistance protein 6**	***Arabidopsis thaliana***	**ABR08780.1**	**3.00E-24**	**94%**	**9.76** ± **0.01**
L115-1b	178	E3 ubiquitin-protein ligase At1g12760-like	*Citrus sinensis*	XP_006476189.1	6.00E-27	96%	0
L247-2b	453	Chorismate synthase/5-enolpyruvylshikimate-3-phosphate phospholyas, putative isoform 2	*Theobroma cacao*	XP_007009819.1	2.00E-61	82%	0.12 ± 0.01
**L202-1a**	**236**	**4-hydroxyphenylpyruvate dioxygenase**	***Arabidopsis thaliana***	**NP_172144.2**	**7.00E-05**	**85%**	**6.23** ± **0.60**
**GENES INVOLVED IN TRANSPORT**							
**L211-3a**	**385**	**Nucleobase-ascorbate transporter 4-like**	***Citrus sinensis***	**XP_006486910.1**	**6.00E-71**	**95%**	+
**L219-1a**	**385**	**Nucleobase-ascorbate transporter 4-like**	***Citrus sinensis***	**XP_006486910.1**	**4.00E-70**	**94%**	+
**L210-1a**	**282**	**Early-responsive to dehydration stress protein (ERD4)**	***Arabidopsis thaliana***	**NP_564354.1**	**7.00E-18**	**59%**	+
L82-3b	296	Heavy metal translocating P-type ATPase	*Gloeocapsa sp. PCC 7428*	WP_015328560.1	1.00E-41	80%	0.35 ± 0.02
L191-1b	246	Protein transport protein Sec61 subunit alpha-like	*Citrus sinensis*	XP_006467607.1	8.00E-41	99%	0
L244-1b	341	BET1P/SFT1P-like protein 14A	*Arabidopsis thaliana*	NP_191376.1	3.00E-45	74%	0
L117-1b	297	Transmembrane emp24 domain-containing protein p24delta9-like	*Cucumis sativus*	XP_004143772.1	7.00E-60	97%	0
L82-1b	121	Ras-related protein RABA2a-like isoform X1	*Citrus sinensis*	XP_006470959.1	1.00E-12	94%	0.32 ± 0.09
L253-9b	121	Ras-related protein RABA2a-like	*Citrus sinensis*	XP_006470954.1	1.00E-12	94%	0.22 ± 0.03
L215-3b	208	RANBP2-like and GRIP domain-containing protein 5/6-like isoform X1	*Citrus sinensis*	XP_006465564.1	8.00E-25	96%	0
**GENES INVOLVED IN SIGNAL TRANSDUCTION**							
**L219-2a**	**238**	**Serine/threonine-protein kinase cx32 isoform 2**	***Theobroma cacao***	**XP_007043058.1**	**8.00E-37**	**90%**	+
**L160-1a**	**253**	**Kinase superfamily protein isoform 1**	***Theobroma cacao***	**XP_007035517.1**	**5.00E-34**	**78%**	+
**L123-2a**	**247**	**Putative dual specificity protein phosphatase DSP8-like isoform X2**	***Citrus sinensis***	**XP_006464442.1**	**1.00E-30**	**76%**	**5.67** ± **0.62**
**L98-4a**	**205**	**Serine phosphatase**	***Anabaena variabilis ATCC 29413***	**WP_011317166.1**	**2.10**	**38%**	**6.89** ± **0.03**
L208-2b	232	Ankyrin repeat-containing protein	*Arabidopsis thaliana*	NP_566360.1	4.00E-26	69%	0
**GENES INVOLVED IN STRESS RESPONSE AND DEFENSE**							
**L177-3a**	**319**	**Putative senescence-associated protein**	***Pyrus communis***	**AAR25995.1**	**2.00E-53**	**99%**	+
L224-2b	314	Plant senescence-associated protein	*Theobroma cacao*	XP_007099561.1	2.00E-40	93%	0.13 ± 0.01
**L65-3a**	**175**	**Phosphoadenosine phosphosulfate reductase family protein**	***Arabidopsis lyrata subsp. lyrata***	**XP_002873090.1**	**2.00E-12**	**56%**	**6.73** ± **0.89**
**L231-2a**	**298**	**Probable glutathione S-transferase-like**	***Citrus sinensis***	**XP_006494151.1**	**1.00E-54**	**94%**	+
**L98-3a**	**273**	**Germin-like protein**	***Arabidopsis thaliana***	**AAB51577.1**	**7.00E-39**	**83%**	**18.22** ± **3.10**
**L235-2a**	**273**	**HSP70-binding protein 1-like**	***Citrus sinensis***	**XP_006480867.1**	**6.00E-49**	**99%**	+
**L119-2a**	**196**	**Protein NDR1-like**	***Cucumis sativus***	**XP_004170854.1**	**3.00E-18**	**95%**	**4.62** ± **0.26**
L220-3b	194	Adenine nucleotide alpha hydrolases-like superfamily protein	*Arabidopsis thaliana*	NP_566564.1	0.002	78%	0.21 ± 0.03
**GENES INVOLVED IN LIPID METABOLISM**							
L198-3b	102	Cytochrome P450 94A1-like	*Citrus sinensis*	XP_006477025.1	3.00E-07	89%	0.45 ± 0.03
L209-4b	205	Cytochrome P450 94A1-like	*Citrus sinensis*	XP_006471355.1	7.00E-35	95%	0.52 ± 0.02
L116-3b	186	Cytochrome P450 86B1-like	*Citrus sinensis*	XP_006484536.1	2.00E-29	96%	0
**L159-4a**	**131**	**Bifunctional epoxide hydrolase 2-like isoform X2**	***Citrus sinensis***	**XP_006479996.1**	**2.00E-15**	**94%**	+
**L239-8a**	**297**	**Probable plastid-lipid-associated protein 14, chloroplastic-like isoform X1**	***Citrus sinensis***	**XP_006490498.1**	**1.00E-56**	**97%**	**22.10** ± **4.60**
**L68-1a**	**314**	**Patatin-like protein 3**	***Phoenix dactylifera***	**XP_008790455.1**	**2.30**	**49%**	+
**CELL WALL MODIFICATION**							
L251-2b	236	Laccase-3-like	*Citrus sinensis*	XP_006488594.1	7.00E-30	82%	0.05 ± 0.01
**OTHERS**							
**L64-2a**	**253**	**Gibberellin 2-beta-dioxygenase 2-like**	***Citrus sinensis***	**XP_006492455.1**	**2.00E-39**	**100%**	+
**L211-2a**	**242**	**Cox19-like CHCH family protein**	***Theobroma cacao***	**XP_007042812.1**	**5.00E-18**	**73%**	+
**L175-3a**	**146**	**Probable S-adenosylmethionine-dependent methyltransferase At5g37990-like**	***Citrus sinensis***	**XP_006494806.1**	**1.00E-12**	**82%**	+
L67-2b	206	Pyridoxal kinase-like isoform X2	*Citrus sinensis*	XP_006482149.1	6.00E-10	97%	0
L116-4b	186	Adiponectin receptor protein 2-like	*Citrus sinensis*	XP_006474059.1	6.00E-28	100%	0.38 ± 0.04
L52-4b	213	Myrcene synthase, chloroplastic-like	*Cucumis sativus*	XP_004150681.1	1.00E-14	51%	0
**L131-1a**	**213**	**Myrcene synthase, chloroplastic-like**	***Cucumis sativus***	**XP_004150681.1**	**1.00E-15**	**53%**	+
L159-3b	200	Calcium-dependent lipid-binding domain-containing plant phosphoribosyltransferase-like protein	*Arabidopsis thaliana*	NP_196801.1	2.00E-22	75%	0.33 ± 0.05
L212-1b	271	Haloacid dehalogenase-like hydrolase family protein isoform 4	*Theobroma cacao*	XP_007012193.1	2.00E-35	75%	0
**L90-1a**	**234**	**Protein gar2-like isoform X1**	***Citrus sinensis***	**XP_006481969.1**	**2.00E-10**	**97%**	+
**L228-1a**	**236**	**Uncharacterized protein LOC102629060 isoform X2**	***Citrus sinensis***	**XP_006469004.1**	**3.00E-39**	**94%**	+
**L175-2a**	**173**	**Hypothetical protein**	***Scytonema hofmanni***	**WP_017746701.1**	**5.00E-07**	**45%**	+
**L141-1a**	**150**	**Hypothetical protein EUTSA_v10008738mg**	***Eutrema salsugineum***	**XP_006416410.1**	**6.70**	**88%**	**9.32** ± **0.14**
**L98-2a**	**288**	**Hypothetical protein CICLE_v10009304mg**	***Citrus clementina***	**XP_006450269.1**	**2.00E-33**	**98%**	**8.98** ± **0.01**
**L199-3a**	**194**	**Hypothetical protein ARALYDRAFT_894413**	***Arabidopsis lyrata subsp. lyrata***	**XP_002888564.1**	**8.00**	**36%**	**5.08** ± **0.46**
**L233-1a**	**328**	**Hypothetical protein CICLE_v10026596mg**	***Citrus clementina***	**XP_006425793.1**	**7.00E-61**	**98%**	+
L99-2b	117	Uncharacterized protein LOC101232191	*Cucumis sativus*	XP_004162796.1	1.00E-07	96%	0.05 ± 0.02
L2-1b	300	Uncharacterized protein LOC102613081 isoform X1	*Citrus sinensis*	XP_006478610.1	2.00E-07	96%	0.18 ± 0.03
L51-1b	227	Hypothetical protein CISIN_1g046391mg	*Citrus sinensis*	KDO35934.1	1.00E-394	99%	0
L224-1b	319	Hypothetical protein	*Arabidopsis thaliana*	BAF01964.1	4.00E-51	100%	0.21 ± 0.03

*Data are means ± SD (n = 3); Expression ratio, 0 means TDFs were only detected in control leaves; + means TDF were only detected in the B-deficient leaves. #, Number; Functional classification was performed based on the information reported for each sequence by The Gene Ontology (http://amigo1.geneontology.org/cgi-bin/amigo/blast.cgi) and Uniprot (http://www.uniprot.org/). Relative expression ratio was obtained by gel image analysis, which was performed with PDQuest version 8.0.1 (Bio-Rad, Hercules, CA, USA). Up-regulated TDFs were highlighted in bold*.

### Validation of CDNA-AFLP data

**Figure 1:**
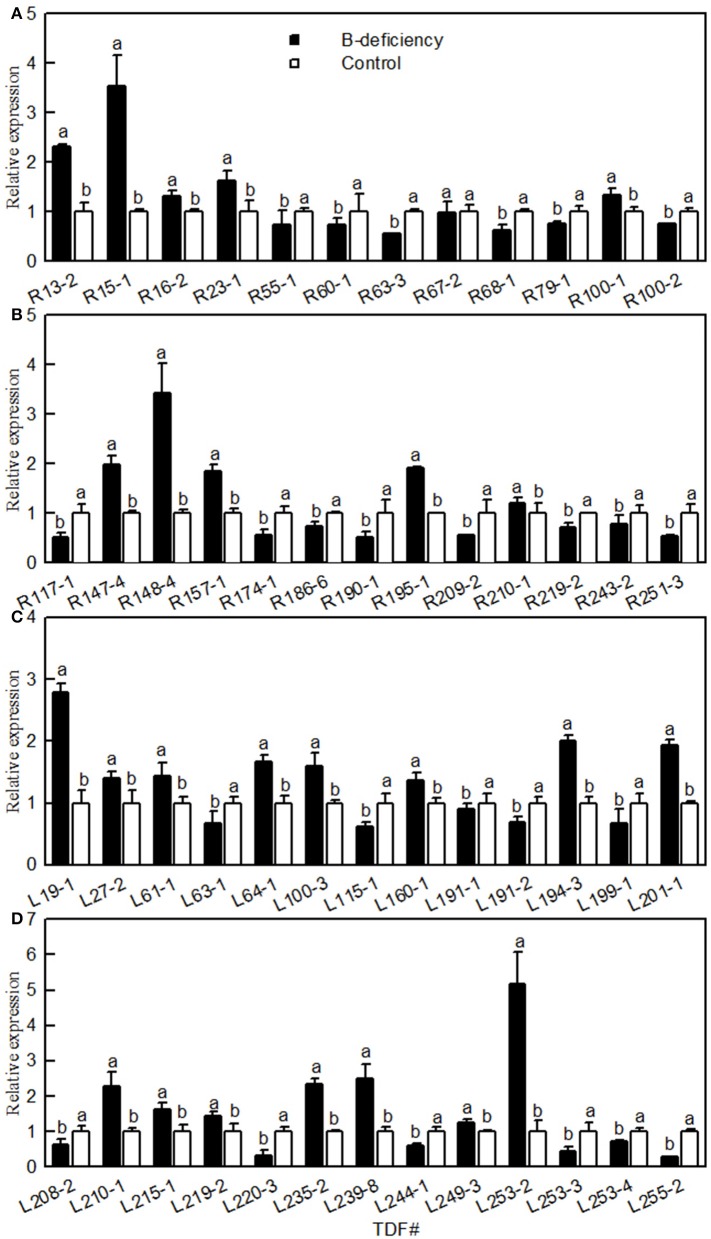
**Effects of B-deficiency on gene expression of ***Citrus sinensis*** roots (A,B) and leaves (C,D)**. qRT-PCR was run in three biological replicates with two technical replicates. For the normalization of gene, citrus *actin* (GU911361.1) was used as an internal standard and the sample from B-sufficient plants was used as reference sample, which was set to 1. Bars represent means ± SD. Different letters above the bars indicate a significant difference at *P* < 0.05.

Twenty-five TDFs from roots and 26 TDFs from leaves were selected for qRT-PCR to check their expression patterns obtained by cDNA-AFLP. The expression profiles of all these TDFs produced by qRT-PCR well-matched with the expression patterns revealed by cDNA-AFLP except for three TDFs (i.e., TDFs #R67-2b, R190-1a and L199-1a; Figure 1). Thus, this technique was validated in 94% of cases.

## Discussion

### B-deficiency-responsive-genes differed between roots and leaves

We isolated less up-regulated TDFs from B-deficient roots than from B-deficient leaves, and more down-regulated TDFs from the former than from the latter (Tables 1, 2; Figure S3). This agrees with our report that mitochondrial respiration, OA metabolism and AA biosynthesis were increased in B-deficient *C. sinensis* leaves with more accumulation of carbohydrates, but decreased in B-deficient *C. sinensis* roots with less accumulation of carbohydrates (Lu et al., 2014a). Furthermore, the vast majority of the differentially expressed TDFs only presented in B-deficient roots or leaves, only seven TDFs [i.e., XP_006484536.1 (TDFs #R118-2b and L116-3b), XP_006488862.1 (TDFs #R100-2b and L100-3a), XP_007042812.1 (TDFs #R211-1b and L211-2a), XP_007043058.1 (TDFs #R219-2b and L219-2a), NP_564354.1 (TDFs #R210-1a and L210-1a), XP_006492455.1 (TDFs #R64-4a and L64-2a) and BAF01964.1 (TDFs #R20-1a, R82-7a and L224-1b)] with the same GenBank ID presented in the both. Except for XP_006484536.1, NP_564354.1, and XP_006492455.1, the remaining four TDFs in roots and leaves displayed different responses to B-deficiency (Tables 1, 2; Figure S3). To conclude, B-deficiency-induced changes in gene expression differed between roots and leaves.

### Genes involved in carbohydrate and energy metabolism

The expression levels of many carbohydrate and energy metabolism-related TDFs were altered in B-deficient roots and leaves (Tables 1, 2; Figure S3). Plant UDP-glycosyltransferases (UGTs) play important parts in enhancing the tolerance of plants to environmental stresses (Bowles et al., 2006). Over-expression of *UGT85A5* conferred salt tolerance in tobacco (Sun et al., 2013). However, over-expression of *UGT73B2* lowered oxidative stress tolerance in *Arabidopsis* (Kim et al., 2010). Our results showed that the expression levels of *UGTs* were decreased in B-deficient roots (i.e., TDFs #R255-1b, R256-2b, R242-1b, R244-2b, and 63-3b) and increased in B-deficient leaves (i.e., TDFs #L201-1a, L3-2a and L29-2a) (Tables 1, 2), which might be related with the less and more accumulation of carbohydrates in B-deficient *C. sinensis* roots and leaves, respectively (Lu et al., 2014a). Thus, we proposed that the adaptive responses of *UGPs* differed between B-deficient roots and leaves.

The expression of *cytochrome P450s* (*CytoP450s*) was increased in *Vicia sativa* seedlings when exposed to plant hormone methyl jasmonate (Pinot et al., 1998) and in B-toxic *C. grandis* leaves (Guo et al., 2014). Transgenic tobacco and potato plants over-expressing *CytoP450* displayed increased monooxygenase activity and enhanced tolerance of oxidative stress after herbicide treatment (Gorinova et al., 2005). Therefore, the up-regulation of *CytoP450* (TDF #R132-6a) in B-deficient roots (Table 1) might contribute to the tolerance of plants to B-deficiency.

Root expression levels of genes related to glycolysis [i.e., *pyruvate kinase* (TDF #R167-3b)] and tricarboxylic acid (TCA) cycle [i.e., *NAD-dependent malic enzyme 59 kDa isoform, mitochondrial* (TDF #R79-1b)] were decreased by B-deficiency (Table 1). This agrees with our report that mitochondrial respiration and OA metabolism were down-regulated in B-deficient *C. sinensis* roots (Lu et al., 2014a). However, B-deficiency induced root expression of *fumarate hydratase 2* (TDF #R59-3a; Table 1). Interestingly, *enolase-like* (i.e., TDFs #L255-2b and L256-2b) associated with glycolysis was repressed in B-deficient leaves (Table 2), which disagrees with our report that B-deficient *C. sinensis* leaves had higher mitochondrial respiration and activities of enzymes in glycolysis and TCA cycle (Lu et al., 2014a).

Hamilton et al. (2001) observed that both root vacuolar ATPase and mitochondrial ATP synthase were induced by aluminum (Al) in an Al-resistant wheat cultivar and suggested that increased ATP synthase activity was required for supporting V-ATPase induction and other energy-dependent processes associated with Al-resistance. Over-expression of a mitochondrial ATP synthase small subunit gene enhanced the salt-tolerance of transgenic tobacco plants (Zhang et al., 2006). Thus, the up-regulation of *ATP synthase subunit* β (i.e., TDFs #R3-1a and R16-2a) in B-deficient roots (Table 1) might be advantage to maintaining energy balance by enhancing ATP biosynthesis, when ATP synthesis was reduced due to decreased root respiration (Lu et al., 2014a).

### Genes involved in nucleic acid metabolism

We isolated eight up-regulated (i.e., TDFs #R23-2a, R15-1a, R198-1a, R13-2a, R157-1a, R105-2a, R195-1a, and R147-4a) and five down-regulated (i.e., TDFs #R81-3b, R157-4b, R247-1b, R164-1b, and R100-3b) nucleic acid metabolism-related TDFs from B-deficient roots (Table 1), which disagrees with our report that the abundances of 60 B-deficiency-responsive protein species associated with nucleic acid metabolism in *C. sinensis* roots were decreased by B-deficiency except for argonaute family protein (Yang et al., 2013b). The difference between the two studies might be caused by post-translational modifications (PTMs).

Heat shock transcription factors (HSFs) play a role in various stresses, including oxidative stress. Davletova et al. (2005) demonstrated that HSFs were indispensable in the early sensing of H_2_O_2_ stress in *Arabidopsis*. Mukhopadhyay et al. (2004) reported that transgenic tobacco plants over-expressing a zinc finger protein (ZFP) gene from rice had enhanced tolerance to cold, dehydration, and salt stress. Our results showed that *HSF24-lik*e (TDF #R23-2a) and *ZFP4* (TDF #R15-1a) were induced in B-deficient roots (Table 1), indicating a possible role of the two genes in B-deficiency-tolerance.

Like roots, 11 leaf TDFs (i.e., TDFs #L19-1a, L67-1a, L194-3a, L253-7a, L235-1a, L116-1a, L184-1a, L253-3b, L175-1b, L239-3b, and L227-1b) associated with nucleic acid metabolism were affected by B-deficiency (Table 2). DEAD box RNA helicases play important roles in plant stress responses. Over-expression of rice *OsBIRH1*, which encodes a functional DEAD-box RNA helicase, in *Arabidopsis* led to up-regulation of defense-related genes and enhanced disease resistance and oxidative stress tolerance (Li et al., 2008). Mishra et al. (2012) showed that WD40 proteins played a key role in stress tolerance of foxtail millet. Transgenic *Arabidopsis* plants over-expressing CCCH-type ZFP gene (*AtSZF1*) were more tolerant to salt stress (Sun et al., 2007). Therefore, the induction of *DEAD box RNA helicases* (i.e., TDFs #L19-1a, L67-1a, L194-3a, L253-7a), *WD40 repeat-containing protein SMU1-like* (TDF #L235-1a) and z*inc finger CCCH domain-containing protein 22* (TDF #L116-1a) in B-deficient leaves (Table 2) might be an adaptive response of plants to B-deficiency.

Plant methyl-CpG-binding domain (MBD) proteins, which control chromatin structure mediated by CpG methylation, play crucial roles in plant development (Grafi et al., 2007). Peng et al. (2006) observed that the *mbd9* mutants had more shoot branches by increasing the outgrowth of axillary buds than wild-type *Arabidopsis*. Our results showed that B-deficiency down-regulated the expression of *MBD-containing protein 9-like isoform X2* (TDF#L175-1b) in leaves (Table 2), indicating that DNA methylation and plant development were probably impaired, thus increasing shoot branching. This agrees with the report that B-deficient plants displayed a relatively weak apical dominance, and a subsequent sprouting of lateral buds (Wang et al., 2006).

Jacobs and Kück (2011) demonstrated that RNA-binding proteins (RBPs) could modulate chloroplast RNA stability and chloroplast splicing, facilitate RNA editing. Lezhneva and Meurer (2004) showed that photosystem I (PSI) function was specifically affected in the high Chl fluorescence (HCF) 145 mutant of *Arabidopsis* due to the lack of the two PSI core proteins. The defect seemed to be a result of increased instability of the tricistronic *psa*A, *psa*B, and *rps*14 transcript (Jacobs and Kück, 2011). We observed that leaf expression of *RBPHCF152* (TDF#L253-3b, Table 2), which functions in the processing of polycistronic chloroplast *psbB-psbT-psbH-petB-petD* transcript, was repressed by B-deficiency. Thus, it is very likely that PSI was impaired in B-deficient leaves, as observed on B-deficient *C. grandis* leaves (Han et al., 2009).

### Genes involved in protein and AA metabolism

In roots, all differentially expressed TDFs [i.e., 60S ribosomal protein L29-1-like (TDF #R100-2b), 60S ribosomal protein L31-like (TDF #R174-1b), elongation factor G (EF-G), chloroplastic-like (TDF #R253-3b) and EF-Tu, chloroplastic-like (TDF #R256-1b)] associated with protein biosynthesis were down-regulated by B-deficiency except for ribosomal protein S3 (TDF #R63-1a) and Asx tRNA synthetase (AspRS/AsnRS) class II core domain-containing protein (TDF #R103-3a) (Table 1). This agrees with our report that the levels of all the differentially expressed ribosomal proteins and EFs were decreased in B-deficient *C. sinensis* roots (Yang et al., 2013b). Therefore, it is likely that protein biosynthesis was impaired in B-deficient roots, thus decreasing root concentration of proteins (Lu et al., 2014a). Similarly, the expression levels of two TDFs [i.e., *F-box protein At4g18380-like* (TDF #R35-2b) and *BTB/POZ domain-containing protein At5g41330-like* (TDF # R112-5b)] related to protein ubiquitination were inhibited in B-deficient roots (Table 1), indicating that the ubiquitination of some proteins might be impaired in these roots.

Unlike roots, we isolated four up-regulated (i.e., TDFs #L132-2a, L100-3a, L209-1a, and L221-1a) and four down-regulated (i.e., TDF #L253-4b, L209-3b, L242-2b, and L256-1b) TDFs related to protein biosynthesis from B-deficient leaves (Table 2). Thus, B-deficiency-induced decrease in leaf concentration of total soluble proteins (Lu et al., 2014a) could not be explained alone by decreased biosynthesis. Our results showed that the expression levels of eight TDFs (i.e., TDFs #L113-1a, L215-1a, L52-3a, L61-1a, L253-2a, L82-2a, L211-1a, and L249-3a) associated with protein ubiquitination and proteolysis was induced in B-deficient leaves. This indicated that protein degradation might be accelerated in B-deficient leaves, thus lowering leaf concentration of total soluble proteins (Lu et al., 2014a).

Plant proteases play crucial roles in keeping strict protein quality control and degrading specific sets of proteins in response to diverse (a)biotic stimuli (García-Lorenzo et al., 2006). FtsH-mediated repairment of the PSII complex thylakoid is required for the light-induced turnover of the PSII D1 protein (Lindahl et al., 2000). Ubiquitin-mediated regulation of protein stability functions in regulating plant responses to abiotic stresses. The substrate-recruiting E3 ubiquitin ligases control numerous cellular processes through influencing the specificity of the ubiquitination pathway (Lyzenga and Stone, 2012). Thus, the up-regulation of eight TDFs (i.e., TDFs #L113-1a, L215-1a, L52-3a, L61-1a, L253-2a, L82-2a, L211-1a, and L249-3a) related to protein degradation in B-deficient leaves might contribute to the B-deficiency tolerance. However, the expression of *E3 ubiquitin-protein ligase At1g12760-lik*e (TDF #L115-1b) was down-regulated in B-deficient leaves (Table 2).

The expression levels of two TDFs (i.e., TDFs #R132-3a and R104-1a) in AA biosynthesis were decreased in B-deficient roots (Table 1), which had less accumulation of total free AAs (Lu et al., 2014a). However, we identified one down-regulated TDF (TDF #L247-2b) in AA biosynthesis and one up-regulated TDF (TDF #L202-1a) in AA degradation in B-deficient leaves (Table 2), which had more accumulation of total free AAs (Lu et al., 2014a).

### Genes involved in cell transport

Membrane traffic not only is a constitutive housekeeping process, but also plays a role in plant tolerance to environmental stresses (Ohno, 2006). The up-regulation of three TDFs [i.e., *putative vacuolar protein sorting-associated protein 13B-like isoform X1* (TDF #R23-1a), *peroxisomal membrane ABC transporter family, PMP family isoform 2* (TDF#R88-6a) and *calcium-binding mitochondrial carrier protein SCaMC-1-like* (TDF#R132-4a)] associated with membrane traffic in B-deficient roots (Table 1) might be an adaptive response to B-deficiency by facilitating the cell transport. Yang et al. (2013b) reported that the abundances of many membrane traffic-related protein species in *C. sinensis* roots were increased by B-deficiency. Besides, the expression of gene encoding early-responsive to dehydration stress protein (ERD4; TDF #R210-1a), a transmembrane (ion channel) protein, was induced in B-deficient roots. However, the expression levels of five transport-related TDFs (i.e., TDFs #R243-1b, R253-2b, R114-1b, R209_2b, and R117-1b) were down-regulated in B-deficient roots (Table 1). Similarly, we obtained three up-regulated (i.e., TDFs #L211-3a, L219-1a, and L210-1a) and seven down-regulated (i.e., TDFs #L82-3b, L191-1b, L244-1b, L117-1b, L82-1b, L253-9b, and L215-3b) transport-related TDFs from B-deficient leaves (Table 2). Obviously, cell transport was affected in B-deficient roots and leaves, thus impairing the transport of some substances, which agrees with the reports that B played a role in cell transport including membrane transport, sugar transport, and hormone transport (Tang and Dela Fuente, 1986; Brown et al., 2002; Zhou et al., 2015).

### Genes involved in signal transduction

Protein phosphorylation and dephosphorylation play a key role in plant stress signal transduction pathways. Transgenic *Arabidopsis* over-expressing *TaSnRK2.4* encoding SNF1-type serine/threonine protein kinase had enhanced tolerance to drought, salt, and freezing stresses (Mao et al., 2010). *Arabidopsis* dual-specificity protein tyrosine phosphatase 2 (AtDsPTP2) plays a role in the tolerance of plants to oxidative stress generated by ozone (Lee and Ellis, 2007). AtPTP1 has been suggested to function in stress responses of higher plants (Xu et al., 1998). Thus, the up-regulation of four TDFs related to phosphorylation (i.e., TDFs #L219-2a and L160-1a) and phosphatases (i.e., TDFs #L123-2a and L98-4a) (Table 2) in B-deficient leaves indicated that protein phosphorylation and dephosphorylation probably played a role in B-deficiency-tolerance.

Unlike leaves, the expression levels of three phosphorylation-related TDFs (i.e., TDFs #R170-1b, R219-2b, and R251-3b) were down-regulated in B-deficient roots (Table 1), indicating that the phosphorylation of some proteins might be impaired in these roots. This agrees with our report that B-deficiency decreased the abundances of protein species in phosphorylation such as protein kinase superfamily protein with octicosapeptide/Phox/Bem1p domain (AT1G79570.1), calcium-dependent protein kinase 21 (AT4G04720.1) and kinase-related protein of unknown function (DUF1296, AT1G29370.1, and AT3G07660.1) in *C. sinensis* roots (Yang et al., 2013b). Also, the expression levels of three TDFs (i.e., TDFs # R119-1b, R243-2b, R182-1b, and R67-2b) in other signal pathway were decreased in B-deficient roots (Table 1). To conclude, signal transduction might be impaired in B-deficient roots.

### Genes involved in stress response and defense

Senescence is a genetically programmed process governed by the developmental age and induced by (a) biotic stresses. The up-regulation of *plant senescence-associated protein* (TDF #R100-1a) and *putative senescence-associated protein* (i.e., TDFs #R148-4a and L177-3a) in B-deficient roots and leaves (Tables 1, 2) indicated that senescence might be accelerated in these tissues. However, the expression of *plant senescence-associated protein* (TDF # L224-2b) was repressed in B-deficient leaves (Table 2).

To deal with oxidative damage caused by reactive oxygen species (ROS), plants have evolved a scavenging system composed of antioxidants and antioxidant enzymes. We found that the expression of TDFs encoding peroxidase (POD) 30-like isoform X2 (TDF #R57-1a), phosphoadenosine phosphosulfate reductase (thioredoxin) family protein (TDF # L65-3a) and probable glutathione glutathione S-transferase (GST)-like (TDF #L231-2a) was induced in B-deficient roots (Table 1) and leaves (Table 2). This agrees with the reports that the total ability of ROS scavenging was enhanced in B-deficient citrus roots (Yang et al., 2013b) and leaves (Han et al., 2008, 2009). However, the expression of three TDFs [i.e., *thioredoxin (Trx) M4, chloroplastic-like isoform X1* (TDF #R186-6b), *thiosulfate sulfurtransferase 18-like isoform X1* (TDF #R178-4b) and s*erine hydroxymethyltransferase 7-like* (TDF #R169-1b)] associated ROS scavenging was inhibited in B-deficient roots (Table 1).

Germin-like proteins (GLPs), which have different enzyme functions, including oxalate oxidase (OXO) and superoxide dismutase (SOD), play a role in plant development and various abiotic stress responses (Dunwell et al., 2008). Transgenic *Arabidopsis* plants overexpressing *Arachis hypogaea GLP2* and *3* displayed enhanced salt-tolerance (Wang et al., 2013). Plant heat shock protein 70s (HSP 70s) function in various cellular processes including protein import into organelles (Shi and Theg, 2010) and folding of *de novo*-synthesized polypeptides (Hartl, 1996). A nuclear-localized HSP70 conferred heat and drought tolerance on tobacco plants (Cho and Choi, 2009). The up-regulation of *GLP* (TDF #L98-3a) and *HSP70-binding protein 1-like* (TDF #L235-2a) in B-deficient leaves demonstrated the possible involvement of the two genes in B-deficiency-tolerance.

Our finding that the expression of *putative disease resistance protein RGA3-like isoform X2* (TDF # R68-1b) and *putative disease resistance protein RGA4-like* (TDF #R112-3b) was repressed in B-deficient roots (Table 1) agrees with our report that four disease resistance genes (i.e., AT3G14460.1, AT5G63020.1, AT4G27190.1, and AT1G12210.1) were down-regulated in B-deficient *C. sinensis* roots (Lu et al., 2014b). Thus, it is likely that the disease resistance was lowered in these roots, which agrees with the report that B improved the disease resistance in plants (Frenkel et al., 2010). However, the expression of *protein nonrace specific disease resistance 1 (NDR1)-like* (TDF # L119-2a) was induced in B-deficient leaves (Table 2).

In conclusion, most of the differentially expressed TDFs in stress defense (tolerance) were up-regulated in B-deficient leaves, while the amount of the down-regulated TDFs was more than that of the up-regulated ones in B-deficient roots (Tables 1, 2; Figure S3). Obviously, great difference existed in B-deficiency-responsive genes related to stress defense between roots and leaves.

### Genes involved in lipid metabolism

Cytochrome P450 86B1 (CYP86B1) participates in the ω-hydroxylation of very long chain fatty acids (FAs), and the biosynthesis of both suberin and polyester monomer (Compagnon et al., 2009). Cytochrome P450 94A1 (CYP94A1), which has FA oxidation activity, functions in cutin biosynthesis (Tijet et al., 1998). Many 3-ketoacyl-CoA synthase (KCS) enzymes take part in FA elongation. In *Arabidopsis*, loss of *KCS20* and *KCS2/DAISY* led to decreased level of total wax in stems and leaves by 20 and 15%, respectively, while an increase of 10–34% was observed in transgenic leaves over-expressing *KCS20* or *KCS2/DAISY* (Lee et al., 2009). The down-regulation of *cytochrome P450 86B1-like* (i.e., TDFs #R118-2b and L116-3b), *cytochrome P450 94A1-like* (i.e., TDFs #L198-3b and TDF#L209-4b) and *KCS 10-like, partial* (TDF #R60-1b) in B-deficient roots and leaves indicated that B-deficiency might impaire FA metabolism and the biosynthesis of related metabolites in these tissues. However, the expression of four TDFs (i.e., TDFs #L159-4a, L239-8a, L68-1a, and R190-1a) associated with FA metabolism was induced in B-deficient roots and leaves (Tables 1, 2). Epoxide hydrolases (EHs) play important roles in cuticle formation, responses to stresses, and pathogen defenses (Morisseau, 2013). Thus, The up-regulation of *bifunctional epoxide hydrolase 2-like isoform X2* (TDF #L159-4a) in B-deficient leaves (Table 2) might contribute to the B-deficiency-tolerance.

### Genes involved in cell wall modification

In cell wall, proline-rich proteins (PRPs) cross-link each other or link to other components (i.e., saccharides and lignin) to form effective protection layer after pathogen infection or wounding (Brisson et al., 1994). Zhou et al. (2015) reported that the expression levels of two PRP2 genes (i.e., JK817586 and JK817604) in B-deficiency-sensitive *Poncirus trifoliate* roots were down-regulated by 24 and/or 6 h B-deficient treatments, while B-deficiency did not alter their expression in B-deficiency-tolerant Carrizo citrange (*C. sinensis* × *P. trifoliata*) roots. Thus, the up-regulation of *14 kDa PRP DC2.15-like* in B-deficient roots might contribute to the B-deficiency-tolerance by reinforcing cell walls.

Expansins, which enable the growing cell wall to extend, are considered to be crucial regulators of wall extension during growth (Link and Cosgrove, 1998). The down-regulation of *expansin-like B1-like* (TDF #R55-1b) in B-deficient roots (Table 1) indicated that root cell elongation might be impaired (Lee and Kende, 2002), thus inhibiting root growth (Figure S1). Similar results have been obtained in B-deficient *Arabidopsis* roots (Camacho-Cristóbal et al., 2008b) and citrus roots (Zhou et al., 2015).

Laccase, which catalyzes the oxidation of phenolic substrates using oxygen as the electron acceptor, is required for lignin polymerization during vascular development in *Arabidopsis* (Zhao et al., 2013). Ranocha et al. (2002) reported that neither lignin level nor composition was affected due to a repression of *laccase* expression in poplar. However, the antisense transgenic population, *lac3AS*, had a two- to three-fold increase in total soluble phenolic level. In addition, *lac3* suppression caused a dramatic alteration of xylem fiber cell walls. We observed that B-deficiency down-regulated the expression level of *laccase-3-like* (TDF #L251-2b) in leaves (Table 2), thus increasing leaf concentration of total soluble phenolics and altering cell wall structure. This agrees with the reports that B-deficient *C. sinensis* leaves had higher level of total phenolics (Lu et al., 2014a) and that B-deficiency altered cell wall structure, thus causing growth defects of *C. sinensis* leaves (Liu et al., 2014).

### Others

*VEP1*, which encodes a short-chain dehydrogenase/reductase with 3-oxo-Δ^4,5^-steroid 5-β-reductase activity in *Arabidopsis*, is required for vascular strand development (Jun et al., 2002). The down-regulation of *3-oxo*-Δ^*4,5*^*-steroid 5*-β*-reductase-like isoform X2* (TDF #R103-1b) in B-deficient roots (Table 1) indicated that B-deficiency might impair root vascular development. This agrees with the report that B-deficiency affected vascular development in roots, stems, leaves, and nodules of plants (Kouchi and Kumazawa, 1975; Bolaños et al., 1994; Hajiboland et al., 2012).

*Gibberellin (GA) 2*-β*-dioxygenase 2-like* (*GA2OX2*; i.e., TDFs #R64-4a and L64-2a) functioned in the catabolism of GAs was induced in B-deficient roots and leaves (Tables 1, 2). This implied that the degradation of GAs might be enhanced in these tissues, thus reducing their concentrations. This agrees with the report that B-deficient plants had lower levels of GAs (Shi and Liu, 2002).

## Conclusions

**Figure 2:**
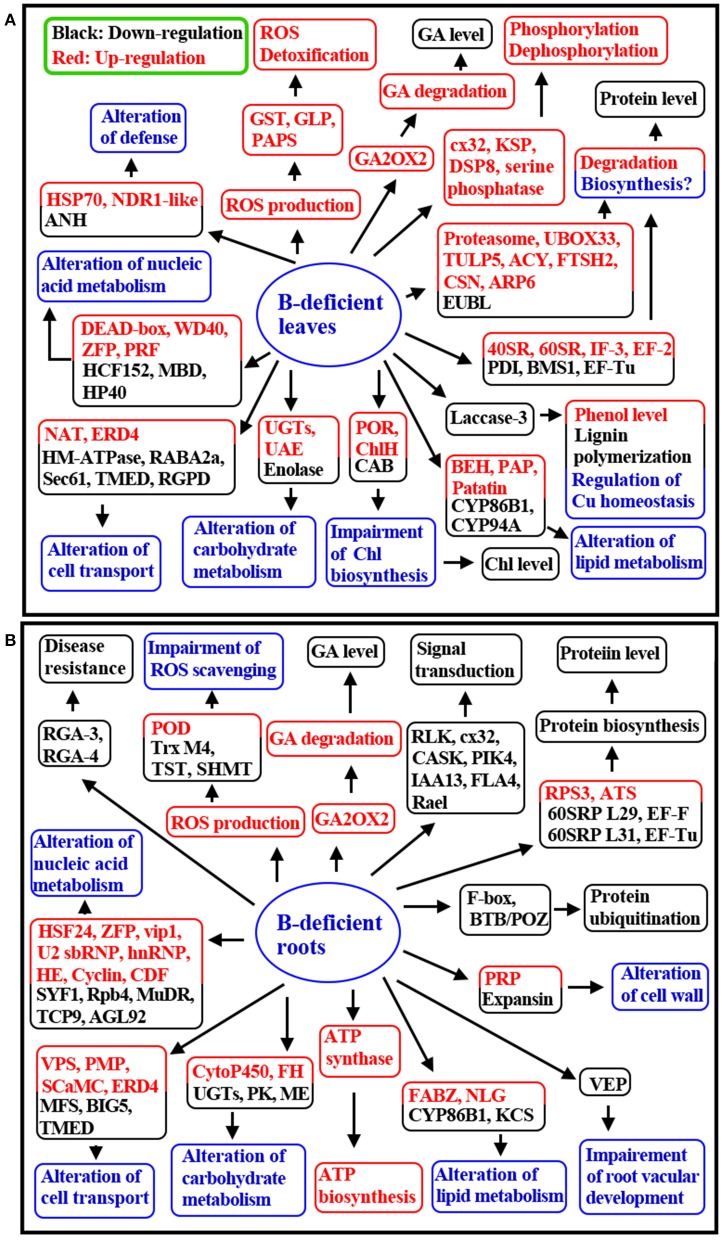
**A proposed model for the responses of ***C. sinensis*** leaves (A) and roots (B) to B-deficiency**. 40SRP, 40S ribosomal protein; 60SRP, 60S ribosomal protein; ACY, Aminoacylase; ANH, Adenine nucleotide α hydrolases-like superfamily protein; ARP6, Auxin-resistance protein 6; ATS, Asx tRNA synthetase (AspRS/AsnRS) class II core domain-contating protein; BEH, Bifunctional epoxide hydrolase; BIG5, Brefeldin A-inhibited guanine nucleotide-exchange protein 5-like; CAB, Chlorophyll a-b binding protein; CASK, Calcium and calcium/calmodulin-dependent serine/threonine-protein kinase; CDF, Cyclin-like family protein; ChlH, Mg-chelatase subunit ChlH, chloroplastic-like; CSN, COP9 signalosome complex subunit 1; cx32, Serine/threonine-protein kinase cx32 isoform 2; DSP8, Putative dual specificity protein phosphatase DSP8-like isoform X2; EF, Elongation factor; EUBL, E3 ubiquitin-protein ligase; FABZ, Beta-hydroxyacyl-ACP dehydratase family protein; FH, Fumarate hydratase 2; FLA4, Fasciclin-like arabinogalactan protein 4-like; FTSH2, ATP-dependent zinc metalloprotease FTSH 2; HE, RRNA intron-encoded homing endonuclease; HM-ATPase, Heavy metal translocating P-type ATPase; hnRNP, Heterogeneous nuclear ribonucleoprotein; HP40, Homeobox protein 40; IF, Translation initiation factor; KSP, Kinase superfamily protein; MFS, Major facilitator superfamily protein; NAT, Nucleobase-ascorbate transporter 4-like; NLG, Non-lysosomal glucosylceramidase-like; PAP, Probable plastid-lipid-associated protein; PAPS, Phosphoadenosine phosphosulfate reductase family protein; PIK4, Phosphatidylinositol 4-kinase; POR, Protochlorophyllide reductase; PRF, Putative replication factor; PRP, Proline-rich protein; RGPD, RANBP2-like and GRIP domain-containing protein; RLK, Receptor-like protein kinase; RPS3, Ribosomal protein S3; SHMT, Serine hydroxymethyltransferas; TMED, Transmembrane emp24 domain; TST, Thiosulfate sulfurtransferase; TULP5, Tubby-like F-box protein 5-like; U2 snRNP, U2 small nuclear ribonucleoprotein; UAE, UDP-arabinose 4-epimerase; UBOX33, U-box domain-containing protein 33; VPS, Vacuolar protein sorting-associated protein.

This is the first comparative investigation of B-deficiency-induced alterations in gene expression profiles in *C. sinensis* roots and leaves using cDNA-AFLP. We isolated more up-regulated TDFs from B-deficient leaves than from B-deficient roots, and less down-regulated TDFs from the former than from the latter, which agrees with our report that mitochondrial respiration, OA metabolism, and AA biosynthesis are enhanced in B-deficient leaves with more accumulation of carbohydrates, but reduced in B-deficient roots with less accumulation of carbohydrates. The majority of B-deficiency-responsive TDFs were isolated only from roots or leaves, only seven TDFs with the same GenBank ID were isolated from the both. Furthermore, only three differentially expressed TDFs shared by the both displayed similar expression trend in response to B-deficiency. Besides, *UGTs* were repressed in B-deficient roots, but were induced in B-deficient leaves; however, TDFs related to ATP biosynthesis were up-regulated in the former, but were unaffected in the latter. Most of the differentially expressed TDFs associated with signal transduction and stress defense were down-regulated in B-deficient roots, but up-regulated in B-deficient leaves. Eight (one) TDFs related to protein ubiquitination and proteolysis were induced (inhibited) in B-deficient leaves, while only two down-regulated TDFs involved in ubiquitination were detected in B-deficient roots. Through integration of the present results and the previous data available in the literatures, we presented a possible model for the responses of *C. sinensis* roots and leaves to B-deficiency (Figure 2). Obviously, many differences existed in long-term B-deficiency-responsive genes between roots and leaves. These findings presented an integrated view of the differential responses occurring in B-deficient roots and leaves and revealed new insight into the different adaptive mechanisms of *C. sinensis* roots and leaves to B-deficiency at the transcriptional level. Our results are also useful for obtaining the key genes responsible for citrus B-deficiency-tolerance and for improving citrus productivity and quality. Therefore, these results are of great importance from the citrus breeding and production point of view.

### Conflict of interest statement

The authors declare that the research was conducted in the absence of any commercial or financial relationships that could be construed as a potential conflict of interest.
