# Supplementary material for: Long-term boron-deficiency-responsive genes revealed by cDNA-AFLP differ between Citrus sinensis roots and leaves
Source: Front Plant Sci. 2015 Jul 28;6:585. doi: 10.3389/fpls.2015.00585 (PMC4517394; doi:10.3389/fpls.2015.00585)
Supplement: Supplementary file 1 [file DataSheet1.DOC]

**Supplementary Material**

**Table S1 | Specific primer pairs used for qRT-PCR expression analysis**.

| **TDF # or Gene** | **Forward primers (5´→3´)** | **Reverse primers (5´→3´)** |
| --- | --- | --- |
| R13-2a | ATCCCCTAACATCCCTTCC | TGCTTCCATCCTTTCCTTG |
| R15-1a | GAGTAATTCGAGGGAAAGAGG | GGAGAACGATTCAGAAGTAAGC |
| R16-2a | CGGATTGTTCACCCACG | CAGCCATTGTCCAGCAGA |
| R23-1a | ACAGGGACAACAAAACCACTAC | CTGTGCCTTCTGTTCCTATTTC |
| R55-1b | AGCTCTGCTGACTTATCTGGAA | GTGGTGACTGACTACGGTGAA |
| R60-1b | TGAAAGAAGATGCAGAGGGG | CTGAGTAAGAGCAAGTTGGTGAAG |
| R63-3b | GGGAGTTCCTTTTCTGTGCT | CAGTGCTTTCACCCGATTAT |
| R67-2b | AGGGTTGGCGTTCATCAT | ATCAGATCCAGCGGTTGC |
| R68-1b | AGCACTGCCCGATCACCTT | CCCCTCCTCTGTTCACCATTC |
| R79-1b | CCAATCGTCTATACCCCAACT | GCTTCCATCCGTAACAACAA |
| R100-1a | ACAATGATAGGAAGAGCCGACA | CGAATACGAACCGTGAAAGC |
| R100-2b | ACAAGGCTCACAAGAACGG | CCCCATAACAGCATCTACTCC |
| R117-1b | GCCCTTGACTGATGAACGC | CAATCCTTAGCAGCCACCC |
| R147-4a | GCATCTCCAGGGTCATCG | TCCTTCCTCCAGGCTTCC |
| R148-4a | ACCGTCGTGAGACAGGTTAGT | TCGGACAGCCGCAAAA |
| R157-1a | GGTACTAATAACCCGGTTGATG | AGGCTTTCCCGTTTGCT |
| R174-1b | ACCCCTTGAGTCCTTCTGG | TCGTCACTGTTGCCGAGAT |
| R186-6b | TGTCACAGCAGGCACTTCA | TCAGACCTGTTCGCTCGTT |
| R190-1a | CATGCTCGTTTCACCACATT | TCTTTGGGTTCAGCGTCC |
| R195-1a | CGAAGGGGAAATGACGGA | TAGGCTTGGCGATGACGGT |
| R209-2b | GTGAAGCTATCGTCAAGCAAAC | CCATCCTACAGCCTCAACCA |
| R210-1a | GATGGTGGTCACTAATAACAAAGA | CAAGAAGCCCAAGGAAGC |
| R219-2b | GAATGGCAGTCCGAGGTAA | TGCAAGTGGCTGAACAGAA |
| R243-2b | GGGAGGGAAGGAGGAAACT | GACTGCCACTGCCATCTGA |
| R251-3b | CACCCAGGTCAATGTCGGA | GCAGATAAGCAATCATGGCAAC |
| L19-1a | GGGCAAGCAACAAGACACC | TTCGGCACGAGGAATGG |
| L27-2a | CTGAAAGAGCCGCCAAGT | TCGCCTGAAGGTATCAACAA |
| L61-1a | CTGTTGGCTGCAAGGAAGA | GGGTGCGGAGGACATTTAG |
| L63-1b | CACCACCGCTTTCCCTT | CCCAATCCCAATGCCTCT |
| L64-1a | CCCTGATGAGCGATTTGAG | CCGCACGGATGTGAGATA |
| L100-3a | GAGTAAAACCCCATAACAGCAT | CCTAAGAAGCACCGCCAC |
| L115-1b | CACCGTTTTCTATTTCTGTCCC | CTCCTGTATTTTGTCCACCTCT |
| L160-1a | AATAGAGGAGCAGGACCGAG | ACTTCCAAAGCCAATGAGACT |
| L191-1b | GCTGATAACCAAGCAAAACAA | GACAACAATAAGGAATACGAGGA |
| L191-2b | GATACTACGATGGACGCTACTGG | CGGACGAATGAATGTGGGT |
| L194-3a | CCCGAGCAAAACAACCC | TCTCCTCACTCAATCCCAACT |
| L199-1a | CGGGCAGTGAAGATGGTT | GGTTTATGTTGGAGGAGTAGGAT |
| L201-1a | TCCAAAACCTGAATAAGCAATA | GAGGGACTAAAGGACCGACA |
| L208-2b | ATGCGGCAAGAGGTGAAC | ACCAGAAGCCAGCAACAAT |
| L210-1a | GATGGTGGTCACTAATAACAAAGA | CAAGAAGCCCAAGGAAGC |
| L215-1a | TTGCCGACAGATGAGAAGG | CCGCACAAATGATTCCAGT |
| L219-2a | GAATGGCAGTCCGAGGTAA | TGCAAGTGGCTGAACAGAA |
| L220-3b | TTCCTCCAGCAAGGGTAAA | AGGGCTATAATGGGCAGTG |
| L235-2a | CAATCGCAGACTGTTGATGTAA | CAGATAGCCAAGAAGAGGAGC |
| L239-8a | CGCTGAGGCTGGCGATAAA | TTGGGGAGCAGGTTGGA |
| L244-1b | TTCCCATCCGTTCCAACA | GGACTACCGTAATAGTAAAGTCGC |
| L249-3a | TTGCTCGGCGGCTTCTT | CCGTAACCATTCCCTCCATC |
| L253-2a | CCAGGATAAACTTGGAGGCA | GAGTAATCGGCTAATCGCTGT |
| L253-3b | GCCTCCGTTGTTACCAAAA | ATCATCCTCCTGTCGTCTCAT |
| L253-4b | GTCCGCCAACGAAAGTGT | GAAGTAAATCGTCGGGAAGC |
| L255-2b | AAGACGACTGGGAGCACTATG | TGAACCTTGCAGACGATGAG |
| *Actin* | AGAACTATGAACTGCCTGATGGC | GCTTGGAGCAAGTGCTGTGATT |

**Figure S1 | Effects of B-deficiency on root (A), stem (B), leaf (C) and whole plant (D) DWs, and B concentration in leaves (E) and roots (F).** Bars represent means ± SD (*n* = 10 except for 6 for leaf and root B concentration). Different letters above the bars indicate a significant difference at *P* < 0.05.


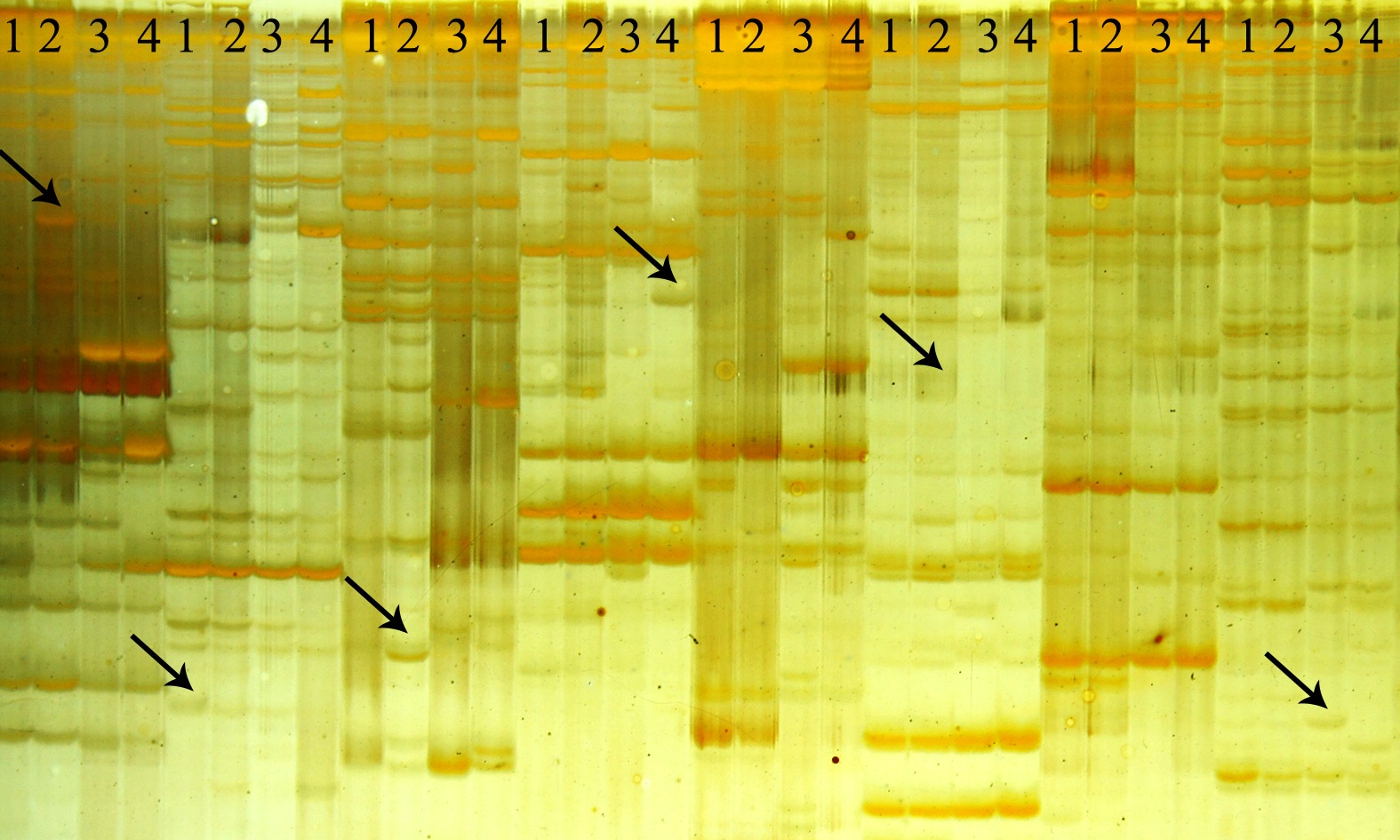


TA/AC TA/AG TA/AT TA/AA TA/CC TA/CG TA/CT TA/CA

**Figure S2 | cDNA-AFLP profiles using one *Eco*R I selective primer and eight *Mes* I selective primers.** One *Eco*R I selective primer: *EcoR* I-TA; Eight *Mes* I selective primers: *Mes* I-AC, AG, AT, AA, CC, CG, CT and CA; 1: B-deficient roots of *Citrus sinensis*; 2: B-sufficient roots of *C. sinenis*; 3: B-deficient leaves of *C. sinensis*; 4: B-sufficient leaves of *C. sinenis*. Arrows indicate differentially expressed TDFs.

**Figure S3 | Differentially expressed TDFs in B-deficient leaves (A) and roots (B) and venn diagram analysis of differentially expressed TDFs identified in B-deficient roots and leaves (C).**
